# The unrealized potential: cohort effects and age-period-cohort analysis

**DOI:** 10.4178/epih.e2017056

**Published:** 2017-12-05

**Authors:** Jongho Heo, Sun-Young Jeon, Chang-Mo Oh, Jongnam Hwang, Juhwan Oh, Youngtae Cho

**Affiliations:** 1 JW LEE Center for Global Medicine, Seoul National University College of Medicine, Seoul, Korea; 2 Center for Healthcare Policy and Research, University of California Davis, Davis, CA, USA; 3 Department of Preventive Medicine, Kyung Hee University School of Medicine, Seoul, Korea; 4 Department of Health Promotion, Daegu University, Korea; 5 Graduate School of Public Health, Seoul National University, Seoul, Korea

**Keywords:** Birth cohort, Cohort effects, Identification problem, Age effects, Period effects

## Abstract

This study aims to provide a systematical introduction of age-period-cohort (APC) analysis to South Korean readers who are unfamiliar with this method (we provide an extended version of this study in Korean). As health data in South Korea has substantially accumulated, population-level studies that explore long-term trends of health status and health inequalities and identify macrosocial determinants of the trends are needed. Analyzing long-term trends requires to discern independent effects of age, period, and cohort using APC analysis. Most existing health and aging literature have used cross-sectional or short-term available panel data to identify age or period effects ignoring cohort effects. This under-use of APC analysis may be attributed to the identification (ID) problem caused by the perfect linear dependency across age, period, and cohort. This study explores recently developed three APC models to address the ID problem and adequately estimate the effects of A-P-C: intrinsic estimator-APC models for tabular age by period data; hierarchical cross-classified random effects models for repeated cross-sectional data; and hierarchical APC-growth curve models for accelerated longitudinal panel data. An analytic exemplar for each model was provided. APC analysis may contribute to identifying biological, historical, and socioeconomic determinants in long-term trends of health status and health inequalities as well as examining Korean’s aging trajectories and temporal trends of period and cohort effects. For designing effective health policies that improve Korean population’s health and reduce health inequalities, it is essential to understand independent effects of the three temporal factors by using the innovative APC models.

## INTRODUCTION

To date, a significant portion of studies on health status or health inequalities in South Korea (hereafter Korea) were cross-sectional studies or studies using short-term accumulated data, which did not consider time lapse. As national-level health data has accumulated during the past decade, it is necessary to examine how population health status and health inequalities change over a longer period, or what are the contributing factors to those changes.

Age, period, and (birth) cohort are demographic concepts that must be considered in the long-term trend analysis on health-related variables of the population. This study aimed to provide a systematical introduction of age-period-cohort (APC) analysis to Korean readers, in particular, health researchers. The analytical method is used to study independent effects of the three factors on long-term trends of health status and health inequalities. Models used in APC studies in Korea were limited to the constrained linear models [1-3] or intrinsic estimators (IE), which will be introduced later [4-8]. In this study, we (1) introduce the concept and effects of cohort, (2) briefly describe the recently developed three innovative APC models, and (3) suggest future research direction. Due to space constraint, this study focuses on explaining the concepts and guidelines for empirical applications of the models, rather than providing the detailed algebraic description of each model. The study also focuses on what questions can be addressed and which analytical method should be applied depending on the type of data.

### Concept and effects of (birth) cohort

A cohort refers to a group of individuals who experience an initial event (e.g., marriage, college entrance) together within a similar period. In medical research, a cohort usually refers to a group recruited as study subjects at the same time point due to their shared clinical characteristics or diseases. In social science including demography or gerontology, a cohort means a birth cohort, a group of individuals born within a specified period. In his classic article [9], Norman Ryder explained that a cohort is defined as people born in a similar time range, entered into the existing social system together, and have similar historical, social, and cultural experiences across life courses. Therefore, cohort effects were generated by the interaction between individuals’ life histories and macro-socioeconomic effects. For example, “the unfortunate class of 1994” in Korea [10] specifically experienced confusions as they were the first generation of the University Scholastic Ability Test and the Academic Divisional System in colleges. As entering the job market during the economic crisis, they also underwent hardships in getting their jobs. Moreover, a more significant portion of the males of the cohort had to serve as active duty soldiers due to the abolition of the Local Soldier System in 1995. Such unfavorable experiences at the critical stages of life would have more considerable adverse effects on their socioeconomic accomplishments throughout their lives, which may bring about distinctive consequences on their health status when comparing to other cohorts.

### Age and period effects

Cohort analysis aims to estimate the effects of cohort on the outcome of interest. It is also called APC analysis [11] because age and period effects should be considered when estimating cohort effects. To date, APC analysis has been widely used to better understand common interests of demographic and epidemiological research, such as morbidity, mortality, and aging. Age effects for epidemiological studies refer to variations caused by the accumulation of age-related or lifecycle-related exposure, a genetic manifestation of diseases, or physiological changes due to natural physical deterioration [12,13]. Period effects are variations in the time periods that affect all population in society regardless of age and cohort at the same time [12], which may reflect the influence of historical events, such as war, labor market situation, macro-economic changes, infectious disease outbreaks, and medical technology development.

Each of these three time-related factors has conceptually independent pathway to affect individuals’ health status or health inequalities in society. Therefore, to understand the long-term changes, it is essential to estimate the independent effects of the three factors simultaneously by using the APC analysis.

## IDENTIFICATION PROBLEM AND HISTORICAL EVOLUTION OF AGE-PERIOD-COHORT ANALYSIS

Since the concept of the cohort has established, researchers have actively explored statistical methodologies to estimate cohort effects. It is a conundrum of APC analysis that the conventional linear regression model fails to discern cohort effects due to the perfect linear-dependent relationship (cohort = period-age) of the three time-related variables. It is also known as “identification (ID) problem.”

Researchers have suggested various analytic methods to solve the ID problem. Two methodologies emerged in the 1970s to 1980s were nonlinear models [14] and constrained generalized linear models [15]. The nonlinear models treat at least one of age, period, and cohort as continuous variables, which is then incorporated into the regression model as quadric, cubic, or higher order functions [16]. Setting one or more variables to have a nonlinear relationship with a dependent variable breaks the perfect linear relationship, and enables to estimate the coefficients of the three time variables. Constrained generalized linear models address the ID problem by having an equality constrain, which is derived from an assumption that effects of two adjacent groups of ages, periods, or cohorts are the same.

Although these two methodologies allowed to estimate coefficient vectors of age, period, and cohort, both methodologies have one crucial limitation: to determine a functional form in a nonlinear model or an equality constraint in a constrained linear model, a researcher should rely on a robust theory or a priori knowledge. That means, it is impossible to estimate the coefficients when such a justification is unavailable. Moreover, as estimates are likely to be sensitive to the selection of the functional form or constraint, a researcher should not arbitrarily select them in any case [17].

Due to the limitations of the previous models, alternative methodologies to address the ID problem have been suggested. In the past decade, a new class of methodologies in the APC analysis that suggest innovative ways to address the ID problem was developed by Yang, Land, and Fu. They argued that the ID problem is a model-specific issue, rather than a data-specific one, and previous models had to deal with the ID problem as they were based on conventional linear regression models. They suggested APC analysis using IE [18]; and the cross-classified random effect model (CCREM) that applies a multi-level analytic framework [19] and the hierarchical APC (HAPC)-growth curve model. These models have been used widely in various academic areas [20-22].

## AGE-PERIOD-COHORT ANALYSIS USING AN INTRINSIC ESTIMATOR

The IE-APC analysis regresses the outcome variable in the nonnull subspace by removing the null space to address the ID problem. The IE-APC analysis employs principal component regression (PCR) techniques. For intuitive interpretation of the coefficients estimated by the PCR using the original scale of A, P, and C, IE-APC transfers them back to the original space (readers can benefit from previous literature for details [12,18]).

**Figure 1.**
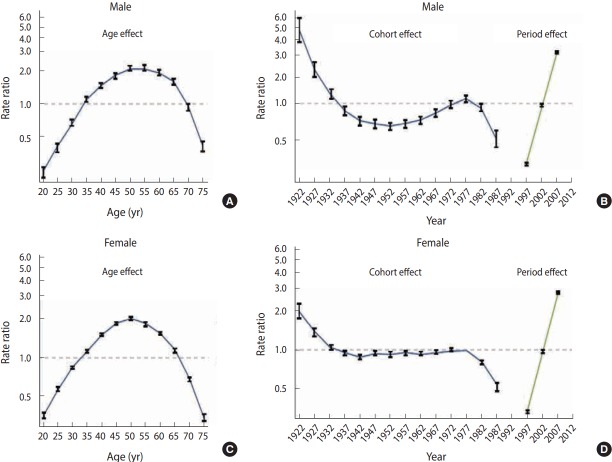
Intrinsic estimator- age-period-cohort analysis of thyroid cancer in Korean adult males (A, B) and females (C, D). From Oh C, et al. Cancer Res Treat 2015;47:362-369 [23].

To better understand the IE-APC analysis, we provide an example of an IE-APC study on thyroid cancer incidence in Korea [23]. Since 1999, the incidence of thyroid cancer rapidly increased in Korea [24], which reached the highest level worldwide [25]. However, the cause of the sudden increase of the thyroid cancer incidence was unclear whether it is due to over-diagnosis along with medical detection technologies or an increase in exposure to other factors, such as ionizing radiation or obesity. In this case, APC analysis may provide a hint of the cause of increased cancer incidence. The result of the IE-APC analysis using 5-year age, period, and cohort thyroid cancer incidence at the population level from 1997 to 2011 was presented in Figure 1 [23]. In males, cohort effects showed a U-shape from those who were born in 1932 to those who were born in 1977; however, no significant changes were observed when compared with period effects. In females, relative risks had no significant changes in those who were born in 1932 and 1977, approaching 1. On the other hand, period effects rapidly increased in both males and females from 1997 to 2011. Subsequently, period effects were significantly associated on the elevation of thyroid cancer incidence in Korea, which suggests that its increase may be not due to changes in external environmental factors or obesity rate, but mostly higher detection rate using ultrasound examinations.

Note that parameter estimators are not always identical with unbiased estimators in IE-APC analysis. Nevertheless, IE-APC has statistically advantageous properties. First, it calculates estimators with better statistical efficiency than previous conventional APC analyses such as constrained generalized linear models [18]. Second, it calculates robust estimators without imposing an additional constraint. Therefore, even when the external information or theory is not established, the model is still estimable [26].

## HIERARCHICAL AGE-PERIOD-COHORT ANALYSIS USING CROSS-CLASSIFIED RANDOM EFFECT MODELS

The models above identified the effects of each A, P, and C based on tabular age by period data at population-level. However, the IE-APC analysis presumes the causes of period and cohort effects; however, an empirical analysis on a specific period or cohort characteristics that contributing the trends cannot be performed. Whereas, the HAPC model allows to have additional covariates of period or cohort characteristics in the model to examine specific causes of long-term health status and inequalities [16,19].

CCREMs are used when two or higher levels have no mutual hierarchical attributes. When CCREMs are applied to APC analysis, effects of age and other individual-level variables are set as fixed effects at level 1, whereas period and cohort effects are set as random effects at level 2 [16,19]. Thus, HAPC analysis can measure random variances of period and cohort effects that were unmeasured at the individual level and can also explain the effects of the period or cohort characteristic variables on trends and changes in the health status and health inequalities [27].

For a better understanding, an exemplar is provided as follows. A researcher identified long-term trends in adult mortality rates based on data collected in a country and then had the following research questions: (1) How did age, period, and cohort effects vary in contributing to the trend of adult mortality rates? (2) Were cohort characteristics, the number of the population born in a similar period (cohort size) and an economic condition at birth, associated with mortality risks? (3) How different are trajectories of mortality rates between males and females as they aged?

**Figure 2.**
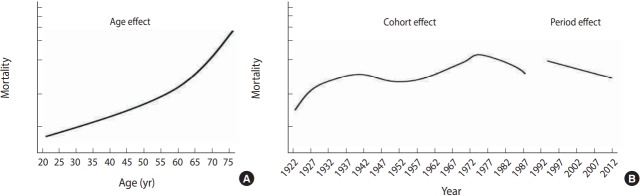
Example of hierarchical age-period-cohort analysis results on adult mortality rate trends (A: age effect, B: cohort effect).

Figure 2 shows age, period, and cohort effects on the mortality between males and females regarding predicted probabilities of mortality. The effect of one factor is mean values when hold effects of the other two factors at their mean values. In other words, age effects at the far left indicate mortality risks at each age group when period and cohort effects were set to their mean values. The graph of age effects shows that the mortality risk exponentially increased with age. When viewed by cohorts, those who were born in the 1970s had the highest mortality risk among all cohorts, and the cohort effects gradually decreased after that. Mortality risk by period effects decreased over time.

For the second research question, both an economic indicator variable of birth years and the cohort size variable linked to the birth year variable were included in the model. In Table 1, the variance of random effects in the first model was 0.345. When the two variables were included in the model, increased mortality risk was significantly associated with the two cohort characteristics. A better economic condition at birth was significantly associated with lower mortality risk later. However, a larger cohort size was significantly associated with increased mortality risk. Economic conditions at birth and cohort sizes explained 60.3% [(0.345-0.137)/ 0.345)*100] and 36.2% [(0.345-0.220)/0.345)*100] of cohort variance, respectively.

**Figure 3.**
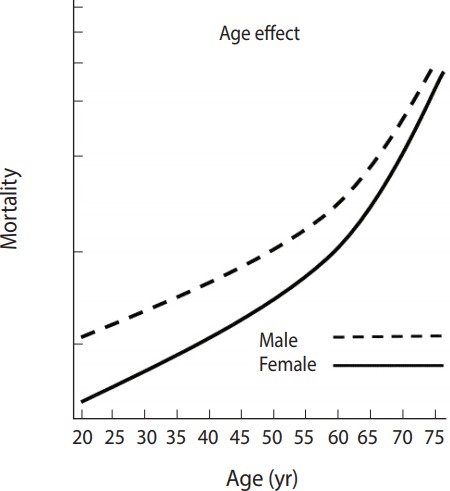
Mortality risk trajectories of males and females by age.

For the answer to the third research question, Figure 3 shows the results after adding the interaction variables between age and sex to the original model. It shows that the trajectories of mortality risk by ages were different depending on sex, indicating that while males had higher mortality risks in all age groups than females, the difference declines with age.

As shown, studies using the HAPC model is capable of considering long-term changes in population composition and in social contexts that determine health status and health inequalities; moreover, it can help reveal the specific determinants of the compositional and contextual effects.

## HIERARCHICAL AGE-PERIOD-COHORT-GROWTH CURVE MODEL USING ACCELERATED LONGITUDINAL PANEL DATA

A growth curve model is used to find intra-cohort and inter-cohort variations in health status with age, which shed light on the mechanism for health inequalities throughout the life cycle. This model can answer the following research questions regarding independent age and cohort effects. First, are there any inter-cohort variations in health trajectories during aging process? In other words, are there cohort differences in age trajectories of health? If yes, the variations by age in the previous aging studies without considering cohort effects can be due to confounding factors between age and cohort effects. Second, are there intra-cohort variations in health trajectories during aging process? This question asks whether age trajectories are different depending on socioeconomic status within each cohort. The first question considers a cohort as a homogeneous group, whereas the second question tries to identify further within-cohort heterogeneity. Third, are there inter-cohort variations within intra-cohort variations in health by age and socioeconomic status? This question aimed to identify intra-cohort and inter-cohort heterogeneities revealing how individuals’ aging has changed according to social, historical, and epidemiologic contexts.

**Figure 4.**
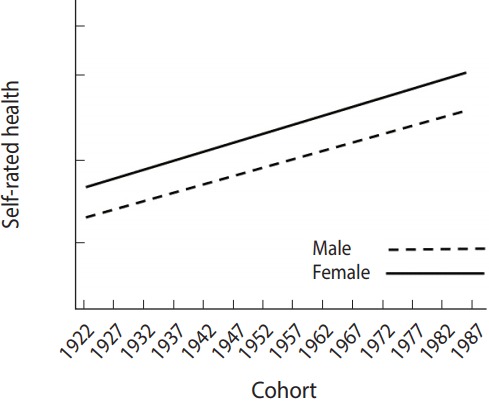
Difference in self-reported health between males and females by cohort.

For a better understanding, an example of the modeling is discussed in Figure 4. The results showed that the mean value of subjective health status increased as cohorts became newer, supporting the hypothesis of inter-cohort variations. In response to the second research question, these results support the hypothesis of intra-cohort inequalities, which demonstrated that continuous inequalities were observed in subjective health status between males and females within cohorts.

**Figure 5.**
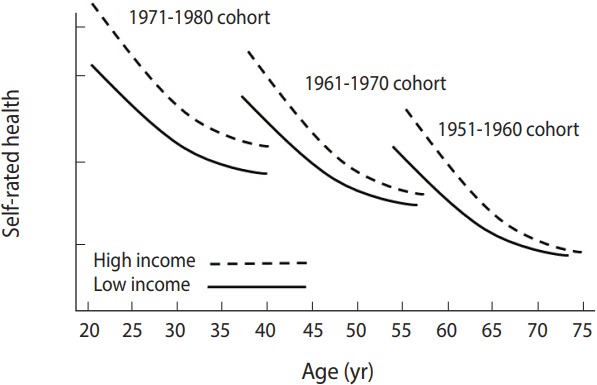
Differences in age growth trajectory of self-reported health by cohort between income groups.

Figure 5 presents an answer to the third research question. To determine whether intra-cohort variations in subjective health by income status are also present between cohorts, a three-way interaction analysis between age, cohort, and income was performed. Figure 5 also shows that changes in inter-cohort differences in subjective health trajectories by income status were altered depending on age. It concluded that growth rates of subjective health by income status were different by cohorts, and the difference got greater.

The limitation of this model is that period effects are not specified. First, this was because a longitudinal study, unlike synthetic cohort data accumulated for a relatively long time, has insufficient study periods. Thus, period effects are considered negligible or omitted for analysis in studies especially for the elderly. Second, this was due to the impossibility of distinguishing period effects in the accelerated longitudinal panel data. In a growth curve model, level 1 analysis can include age or period depending on a research question; however, age and period are the same for an individual, so that the two aspects cannot be considered simultaneously.

Along with accumulation of high-quality data and advancement of analytical models, APC methodologies have been being developed to address the ID problem in more innovative ways. Meanwhile, as some researchers report inconsistent estimates of the APC methodologies, the debate on whether the ID problem can be solved or not is still ongoing. As most scholars agree that there is no “magic bullet” model in the APC analyses due to the ID problem, the methodologies presented above should also be carefully applied with a sufficient understanding on the given data and models.

## CONCLUSION: FUTURE OF AGE-PERIOD-COHORT STUDY IN KOREA

Mannheim [28] claimed that if a society experiences rapid changes, generation gaps will become wide because differences in inter-cohort historical and cultural experiences are substantial. In analytical perspectives, such societies may have a more significant deviation in cohort effects than other societies. Korea underwent short-term but intense socioeconomic changes after the liberation from the Japanese occupation during the World War II. Thus, APC studies will reveal how distinct are the trajectories of Korean aging, how period and cohort effects have been changed, and what are the biological, historical, and socio-structural factors that contribute to health status and inequalities throughout the life cycle of the Korean population.

Several national-level health data in Korea are available for the APC methodologies. Data for an IE-APC analysis on incidence or mortality rates due to chronic diseases, including cancers can be obtained from the Korean Statistical Information Service (https://kosis.kr). The HAPC-CCREM analysis can be applied to representative repeated cross-sectional data such as the Korea National Health and Nutrition Examination Survey (started in 1998) and the Community Health Survey (started in 2008). For an HAPC-growth curve analysis, data are available from the Korean Welfare Panel (started in 2006), the Korea Health Panel (started in 2008), and the Korean Longitudinal Study of Ageing (started in 2006), as well as large-scale database provided by the National Health Insurance Service and the Health Insurance Review & Assessment Service. By utilizing the APC analyses, researchers can analyze long-term trends of health status, health behaviors, health inequalities, aging, chronic diseases, and life cycle.

Furthermore, social and health policies should be specified for dimensions of not only period or age but also cohort. For example, policies may be required based on the cohort size to predict service demands of education, healthcare, and welfare areas to allocate resources. Cohort-specific policies, such as additional childbirth and rearing supports in the early life cycle during economic recessions, may be useful.

## Figures and Tables

**Table 1.** Estimates and variances of cohort-specific variables

	β	Variance
Covariate adjusted	-	0.345
GDP at birth	-0.458^***^	0.137
Cohort size	0.598^***^	0.22

GDP, gross domestic product.

*** p<0.001.
