# Supplementary material for: The unrealized potential: cohort effects and age-period-cohort analysis
Source: Epidemiol Health. 2017 Dec 5;39:e2017056. doi: 10.4178/epih.e2017056 (PMC5790985; doi:10.4178/epih.e2017056)
Supplement: Supplementary file 1 [file epih-39-e2017056-supplementary.pdf]

Volume: 39, Article ID: e2017056
https://doi.org/10.4178/epih.e2017056

출생 코호트 효과와 연령-기간-코호트 분석

The Unrealized Potential: Cohort effects and Age-Period-Cohort Analysis.

허종호 [1], 전선영 [2], 오창모 [3], 황종남 [4], 오주환 [1], 조영태 [5*]

[1]서울대학교 의과대학 이종욱글로벌의학센터, [2]캘리포니아 주립대학교 데이비스 캠퍼스 건강정책 연구소, [3]경희대학교 의학과, [4]대구대학교 건강증진학과, [5]서울대학교 보건대학원 보건학과

Objectives: This study aims to provide a systematical introduction of age-period-cohort (APC) analysis to South Korean readers who are unfamiliar with this method (we provide an extended version of this study in Korean). As health data in South Korea has substantially accumulated, population-level studies that explore long-term trends of health status and health inequalities and identify macrosocial determinants of the trends are needed. Analyzing long-term trends requires to discern the independent effects of age, period, and cohort using APC analysis. Most existing health and aging literature have used cross-sectional or short-term available panel data to identify age or period effects ignoring cohort effects. This under-use of APC analysis may be attributed to the identification (ID) problem caused by the perfect linear dependency across age, period, and cohort.

Methods: This study explores recently developed three APC models to address the ID problem and adequately estimate the effects of A-P-C: (1) intrinsic estimator-APC models for tabular age by period data; (2) hierarchical cross-classified random effects models for repeated cross-sectional data; and (3) hierarchical APC-growth curve models for accelerated longitudinal panel data. An analytic exemplar for each model was provided.

Results: APC analysis may contribute to identifying biological, historical, and socioeconomic determinants in long-term trends of health status and health inequalities as well as examining South Korean's aging trajectories and temporal trends of period and cohort effects.

Conclusion: For designing effective health policies that improve Korean population's health and reduce health inequalities, it is essential to understand the independent effects of the three temporal factors by using the innovative APC models.

Keywords: birth cohort, cohort effects, identification problem, age effects, period effects

I. 도입

그간 국내 건강 또는 건강불평등 연구는 시간의 흐름을 고려하지 않은 단면 연구 내지는 단기간 축적된 자료를 사용한 연구가 많이 이루어졌다. 최근 들어 국가단위의 보건의료 데이터들이 10 년 안팎으로 축적되어 감에 따라 인구집단 건강 수준 및 건강불평등의 장기적인 추세를 관찰하거나 이에 기여하는 요인들의 효과를 추정하려는 연구가 가능해지고 있다.

이러한 인구집단의 건강관련 결과 변수에 대한 장기적 추세 분석을 실시할 때 반드시 고려되어야 하는 인구학적 개념이 있는데 바로 연령, 기간, (출생)코호트이다. 개인수준에서 보면 사람은 시간에 따라 성장과 쇠약, 건강의 악화와 회복 및 증진을 경험하면서 장기적으로는 나이가 듦에 따라 건강이 악화되는 추세를 보인다. 사회적 관점에서 보면 과거 세대가 퇴장하고 새로운 세대가 태어나면서 인구집단의 구성이 계속 변하는 동시에 역동적인 사회경제적 변화도 지속적으로 일어나면서 인구집단의 건강수준과 건강불평등이 변동한다. 본 연구는 이러한 요인들 각각의 독립적인 효과가 건강수준 및 건강불평등의 장기적인 추이에 미치는 영향을 분해하는 방법인 연령-기간-코호트 분석법(Age-Period-Cohort analysis, 이하 APC 분석법)을 국내 보건의료 연구자들에게 체계적으로 소개하고자 한다. 그간 국내에서 APC 분석법을 사용한 논문은 제약적선형모델[1-3] 또는 추후 소개할 고유추정치를 이용한 APC 분석법에 국한되어 왔다[4-8]. 이에에 최초로 소개하고자 한다. 본 논문은 (1) 먼저 코호트의 개념 및 효과에 대해 소개한 후 (2) 이를 분석하기 위해 최근에 개발된 세가지 APC 분석법을 간략하게 소개하고 (3) 향후 국내 연구 방향을 제시하였다. 지면의 제한으로 인해 각 모델의 수식과 자세한 분석방법 대신 관련 개념을 설명하고 어떠한 연구 질문에 답할 수 있는 분석방법인지, 또 자료의 형태에 따라 어떤 분석법이 적용되어야 하는지에 대한 설명에 중점을 두었다.

1. (출생)코호트의 개념 및 코호트효과

코호트는 어떤 일을 함께 시작한 집단이라는 개념으로 같은 시기의 결혼 또는 대학입학 등 정의에 따라 여러 의미를 갖는다. 보건의학연구에서 코호트는 대체로연구의 주제가 되는 특성이나 질환을 공유함으로써 같은 시기에 연구대상으로 모집된 집단을 의미하지만, 인구학과 고령연구를 비롯한 사회과학에서 코호트라 하면 일반적으로 특정 기간 내에 태어난 개인들의 집단인 출생 코호트를 의미한다(본 논문에서도 “코호트”는 출생 코호트를 의미함). 세대(generation)에 대한 사회학적 개념을 확립한 칼 만하임은 세대 문제, 세대 갈등에 대한 그의 사회학적 고전[9]에서 코호트의 개념을 처음 도입하였다. 이후 코호트의 개념을 더욱 확장하여 사회과학에서 자리잡도록 기여한 사람은 노먼 라이더(Norman Ryder)이다. 그는 자신의 유명한 논문[10]에서 코호트와 관련해서 세 가지를 언급했다. 첫째, 코호트는 비슷한 시기에 태어나서 기존의 사회적 체계 안으로 들어와서 비슷한 생애주기 단계에 비슷한 역사적 · 사회적 · 문화적 경험을 하게 된다. 따라서 코호트효과는 개인의 생애사와 거시적 사회경제적 영향력과의 교차로 인해 형성된 경험을 반영한다. 예를 들면, 국내에서 한동안 “비운의 94 학번”이라는 말들이 회자되던 적이 있었다[11]. 94 학번은 수능 첫 세대로서 대입의 혼란을 겪었고 대입 후에는 처음 시행되는 대학 학부제를 겪었으며 95 년에 방위제도가 없어지면서 현역 근무자가 늘었고 취업을 앞두고 IMF 사태를 겪었다. 생애주기의

주요한 단계에서의 불리한 경험들은 장래 이들의 사회경제적 성취, 나아가 건강수준에도 악영향을 미치게 될 것이며, 다른 코호트들의 그것들과의 차이를 만들어 낼 것이다. 두 번째, 라이더는 코호트를 사회적 변화의 핵심 동력으로 보았다. 다른 경험을 가진 후대 코호트들이 계속 사회를 이어가면서 인구의 구성이 바뀌는 것을 인구학적 대사작용(demographic metabolism)이라고 명하고 이를 통해 사회가 변혁된다고 보았다. 세 번째, 그는 인구집단의 행동양식을 결정짓는데 있어서 코호트의 개념이 사회경제적 지위(SES)의 개념만큼 중요한 사회적 구조라고 주장했다. 따라서 코호트의 개념은 건강 수준 및 건강불평등의 역사상 추세와 변동을 결정짓는 사회구조의 한 측면으로 인식될 필요가 있다.

2. 연령효과, 기간효과, 코호트효과

코호트효과를 분석하는 방법을 코호트 분석법(cohort analysis)라고 하는데 실제 코호트의 효과를 분석하기 위해서는 반드시 연령효과(age effects)와 기간효과(period effects)를 동시에 고려한 분석을 해야 하기 때문에 APC 분석법으로 불리우기도 한다[12]. APC 분석법은 인구학과 역학 분야에서 각각 독립적으로 발전되어 왔는데 주로 건강, 사망, 노화와 관련된 공통의 관심분야에 사용되어 왔다. 각 효과의 정의를 살펴보면, 연령효과란 역학 연구에 있어서 나이와 연관된 노출의 축적, 질병의 유전적 발현, 또는 자연적인 육체의 노쇠로 인한 생리학적 변화로 인한 변이를 의미한다[13]. 연령효과는 또한 생애주기 단계에 따른 생리학적 변화와도 연관 지을 수 있다[14]. 기간효과는 연령과 상관없이 한 사회를 구성하는 모든 인구집단에 동시에 영향을 미치는 특정 시간과 관련된 변이를 의미한다[13]. 예를 들어, 전쟁과 같은 역사적인 일들, 노동시장 상황, 거시경제적 변동, 감염병의 창궐, 의학적 기술의 확산과 같은 것들이 있을 수 있다. 이 세 가지 효과의 이해를 돕기 위해 Suzuki[15]의 내용을 재구성한 다음과 같은 대화를 생각해보자.

직장상사: 요즘 피로가 쉽게 가시지 않아. 나이가 들었나 봐. [연령효과]

부하직원: 스트레스 때문 아닐까요? 올해 실적목표 달성 때문에 더 피곤하신가 봐요. 저도 그 때문에 만성피로에 걸린 듯... [기간효과]

직장상사: 얼씨구. 하여간 요즘 애들은 너무 부실하다니까! [코호트효과]

한편, 연령효과, 기간효과, 코호트효과는 하위 인구집단의 사회 경제학적 특성에 따라 그 효과가 달라질 수 있다. 다시 말해, 같은 나이, 같은 코호트 집단의 구성원들 사이에서도 그들이 살아온 삶의 사회경제적 수준 등에 따라 연령효과와 코호트효과의 편차가 발생할 수 있는 것이다. 예를 들면 경제위기와 같은 기간효과는 낮은 교육수준의 노동자들 또는 비정규직 근로자들에게 더 큰 피해를 줄 수 있다. 나아가 세 가지 효과들 간의 상호작용도 고려해볼 수 있다. 예를 들어, 경제위기라는 기간효과는 당시 취업, 은퇴를 앞둔 코호트 집단에 더 큰 악영향을 미칠 가능성이 있다.

건강수준 또는 건강불평등의 장기간의 변동을 분석한다고 할 때 세 가지 효과를 구분하되 동시에 고려하지 않으면 장기간 변동에 기여한 세 가지 효과에 대한 의미 있는 추정이 불가능하다. 이는 II 장에서 자세히 다루고자 한다.

Volume: 39, Article ID: e2017056
https://doi.org/10.4178/epih.e2017056

3. APC 분석법의 유용성

APC 분석법은 건강 및 건강불평등 연구에 주로 사용되어 온 연구방법들과 비교할 때 크게 세 가지의 장점을 가지고 있다. 첫째는 질병의 발생율 또는 사망률의 추세 분석에 유용하다. 기존의 추세 연구는 대부분 연령보정법을 사용하는데 이는 존재가능성이 농후한 코호트효과를 무시한 분석법이다. 연령보정은 연령 기준에 의존한 방법인데, 문제는 연령기준이 시간에 따라 변한다는 것이다. 따라서 Williams 가 언급한대로 "연령보정법은 비교를 위한 상대적인 지표이고 위험에 대한 실제적인 측정법은 아니다[16]." 따라서 코호트효과와 기간효과를 동시에 고려하지 않을 경우, 연령군별 발생률 혹은 사망률이 왜곡되어 측정될 위험이 있다[17]. 또한, 두 사회의 발생률 또는 사망률의 추세가 유사할 경우에도 주요 기저 원인은 다를 수 있는데, 이를 간과할 수 있다[18, 19].

둘째, 고령 연구에 유용하다. 고령화 및 생애주기 연구의 핵심 질문 중의 하나는 "어떠한 인구집단의 건강이 연령과 생애주기에 따라 어떻게 변동하는가"이다. 그런데 기존의 분석 접근법은 연령효과와 다른 두 효과와의 교란(confounding)을 통제할 수 없다. 예를 들어, 중년층과 고령층 간에 음주량에 대한 연령효과를 측정하기 위해서 단면연구를 하게 되면 나이 변수의 계수는 나이효과 및 코호트효과가 혼재되어 있어서 정확히 연령효과를 파악하기 불가능하다. 인구집단의 구성효과를 통제하기 위해 패널연구를 통해 1980 년에 45 세인 사람들의 음주량을 2000 년 65 세의 나이에 다시 측정하였다고 가정해보자. 그럼에도 불구하고 이 20 년의 차이로 인한 효과가 연령효과인지 기간효과인지 명확히 분간할 수가 없다. 이에 비해 APC 분석법은 장기간 축적된 건강관련 데이터를 사용하여 이에 대한 답을 할 수 있다.

세 번째 장점은 APC 분석법이 인구집단의 건강과 건강불평등의 장기간 추세를 둘러싼 여러 맥락들, 즉 역사적 · 사회적 · 문화적 · 정치적 요인의 영향을 이해하는 데 도움이 된다는 점이다. 특히, 코호트 특성과 관련된 요인들의 영향 분석은 APC 분석으로 독특하게 보여줄 수 있는 장점이다. 코호트효과는 생애 초기의 노출과 그 이후 노출의 지속적인 축적이라는 두 가지의 효과로 인해 코호트간에 건강과 사망 위험에 있어서 차이를 가져온다[20]. 따라서 APC 분석법을 사용하여 생애 초기의 부정적 노출 요인이 무엇이고 생애 단계에 걸친 맥락적 노출 요인은 무엇인지 밝힐 수 있다. 생애 초기의 부정적 노출 요인을 밝히려는 연구는 태아기원가설(fetal origin hypothesis)을 근거로 이루어져왔는데, 태아나 신생아 시기의 영양실조 등의 부정적인 영향이 출생 전 혹은 생애초기의 성장에 악영향을 미치게 되고 이것이 성인이 되어 심혈관질환, 호흡기질환, 암, 대사증후군, 사망률 위험을 높이는 것으로 나타났다[21]. 기술생리혁명이론(the theory of technophysio evolution)도 20 세기에 걸쳐 개인의 건강자본이 코호트에 따라 변화되어 왔다고 설명하며 뒷받침하고 있다[22]. 즉, 최근의 코호트 일수록 생애초기부터 훨씬 나은 생리적인 역량을 부여 받게 되었을 뿐만 아니라, 의학기술의 발전으로 연령에 따른 건강자본의 감소율도 낮아지는 것을 보여주었다. 이것은 최근의 코호트 일수록 만성질환 및 장애의 시작이 늦어지고 있다는 선행 연구와 일맥상통하고 있다[23, 24]. 이러한 코호트 내의 동질적, 코호트 간의 이질적 특성이 그 외의 각종 건강 및 건강불평등 관련 결과변수의 장기간의 추세에도 영향을 미칠 것은 충분히 예상 가능하다. 따라서 APC 분석법은건강수준 및 건강불평등의 장기간에 걸친 인구집단의 구성 변화와 사회맥락의 변화를 동시에 고려하여 측정할 수 있을 뿐 아니라 건강 및 건강불평등을 결정하는 해당 구성효과와 맥락효과의 요인이 무엇인지 밝히는데 도움이 될 수 있다.

Volume: 39, Article ID: e2017056
https://doi.org/10.4178/epih.e2017056

II. 식별문제(identification problem)와 이를 해결하기 위한 APC 분석법의 역사적 진화

코호트의 개념이 정립된 이래, 코호트효과를 양적으로 측정하는 통계적 방법론에 대한 연구들도 활발히 진행되어 왔다. 코호트가 종속변수에 미치는 고유한 효과를 측정하기 위해서는 다른 두 시간 변수들(연령, 기간)이 종속변수에 미치는 영향을 측정하여 코호트효과에서 통계적으로 분리시키는 것이 필수적이다. 일반적으로 이러한 분석을 하기 위해서는 다중회귀모델(multiple regression) 이 사용된다. 예를 들면, 연령, 기간, 코호트가 독립변수로 포함된 다음과 같은 모델을 생각해볼 수 있다.

$$Y = \alpha + \beta_1 \text{연령} + \beta_2 \text{기간} + \beta_3 \text{코호트} + \varepsilon \qquad \text{(II−1)}$$

여기서, Y 는 종속변수; α는 절편; β_1, β_2, 와 β_3 는 각각 연령, 기간, 코호트의 편회귀 기울기; 그리고 ε는 랜덤오차이다 [25]. 이 모델의 편회귀 기울기를 측정하면, 그 측정값을 각 변수가 종속변수에 미치는 독립적 효과로 해석할 수 있다. 그러나 APC 분석법의 경우 세 독립변수가 갖는 완벽한 선형적 의존관계(코호트=기간−연령)로 인해 선형회귀모델의 설계행렬(design matrix)은 특이행렬(singular matrix)이 되어[26], 최소제곱법(ordinary least square)이나 최대가능도법(maximum likelihood)으로는 편회귀 기울기의 계수벡터를 측정할 수 없다.[1] 이는 APC 분석의 난제로, 식별문제(identification problem)라고도 불린다.

계수벡터를 측정하기 위해 식별문제를 어떻게 다룰 것인지에 대해, 그간 활발한 연구들이 이루어졌다. 1970 년에서 1980 년대에 걸쳐 대두된 두 가지 방법론은 비선형모델(nonlinear model[27])과 제약된 일반선형모델(the constrained generalized linear model[25])이다. 비선형 모델은, 독립변수인 연령, 기간, 코호트 중 하나 이상을 연속변수로 사용하여, 2 차나 3 차, 혹은 그 이상의 고차 함수로 회귀모델에 포함시킨다[28]. 즉, 하나 이상의 변수를 종속변수와 비선형 관계를 갖게 함으로써, 선형회귀모델에서 종속변수와 세 시간 변수가 갖는 완벽한 선형 관계를 깨고, (II−1)을 측정 가능한 회귀 모델로 변형하는 것이다. 제약된 일반선형모델의 경우, (II−1)의 선형회귀분석모델을 유지하되, 추가로 제약조건 부과함으로써, 각 효과를 측정 가능하도록 유도하는 방법이다. 제약조건으로는, 이웃하는 두 연령, 기간 혹은 코호트 그룹의 효과가 동일하다고 가정하여 균등제약(equality constraint)을 부과하는 것이 일반적이다($\beta_n = \beta_{n+1}$).

이상의 두 방법론은 연령, 기간, 코호트의 계수 벡터를 측정해낸다는 점에서 APC 분석법의 유의미한 진보를 이뤄냈으나, 여전히 방법론적 한계를 지닌다. 바로 비선형 모델에서의 구체적 함수 형태나, 제약적선형모델에서의 균등제약은 임의적으로 지정할 수 없기 때문에 정립된 이론이나 선험 연구에 근거를 두고 설정되어야 한다는 점이다[29]. 특히, Yang 과 그의 동료들은 연령, 기간, 코호트효과의 측정값은 함수 형태나 균등제약에 의해 매우 민감하게 변한다는 연구 결과를 발표했는데[30], 이 때문에 이론이나 선험 연구가 존재하지 않거나 아직 확립되지 않은 경우, 여전히 모델이 측정 불가능하거나, 임의적인 함수 형태나 균등제약이 선택됨으로써 편향된 추정치를 얻을 위험이 있다.

---

[1] 선형회귀모델의 에러텀이 정규분포를 따르는 경우, 최소 제곱법과 최대 가능도법에 의한 계수벡터의 추정량 (estimator)은 $(X^TX)^{-1}X^TY$ 로 동일하다. 설계행렬 X 가 특이행렬인 경우, $(X^TX)^{-1}$ 를 구하는 것이 수학적으로 불가능하여 무수한 계수 벡터를 근으로 갖게 되고, 그 중 어떤 것이 주어진 데이터를 생성한 연령, 기간, 코호트 효과인지 식별할 수 없다.

https://doi.org/10.4178/epih.e2017056

기존 모델의 이러한 한계 때문에, 식별의 문제와 대안적 방법론에 대한 연구는 후에도 계속 되어왔다.새롭게 연구된 통계학적 모델들이 식별의 문제를 해결하기 위해 APC 분석에도 적용되었는데, 예를 들면, 부분최소자승법(partial least square regression)[31], 일반역행렬(generalized inverses)[32]나, 근사곡선(approximating splines)[33]과 같은 고급 통계기술을 이용한 방법론들이 발표된 바 있다. 2000 년 후반에 통계적 발전에 힘입어 새로운 방법들이 Yang C. Yang, Kenneth Land, Wenjiang Fu 와 같은 연구자들을 중심으로 이전의 방법들과는 새로운 단계의 연구 모델이 개발되었다. 기존 연구들은 선형 모델을 사용하여 식별 문제가 APC 분석법의 해결할 수 없는 문제라고 결론 내린 반면, 식별 문제가 데이터 특성에 달린 것이 아니라 모델 특성에 따라 다르다는 것에 착안하여 나이, 기간, 코호트를 덧셈고정효과모델(additive fixed effects model)의 독립적인 공변량으로 취급하지 않는 방법을 사용한 고유추정치(intrinsic estimator)를 이용한 APC 분석법[34]과 다수준 분석 관점을 도입한 교차분류임의효과 모델(cross-classified random effect model)[35]과 위계적 APC-성장곡선모델(Hierarchical APC-growth curve model)이다. 해외의 경우 해당 모델들을 적용한 다양한 연구가 이루어지고 있다[36-38].

III. 고유추정치(intrinsic estimator)를 이용한 APC 분석법(IE-APC 분석법)

IE-APC 분석법은 식별의 문제를 해결하기 위해 주성분분석(Principal Component Analysis) 기술인 주성분회귀분석을 이용한다[30, 31]. 주성분분석은 원래의 설명변수 대신 가상의 주성분으로 대체하는 방법으로, 추정된 주성분들은 선형결합상 서로 직교하기 때문에, 각 주성분끼리는 서로 독립이라 할 수 있다. 주성분회귀분석은 원래의 회귀방정식에서 고유값 분해를 통해 설명변수들의 선형결합으로 이루어진 주성분들을 설명변수의 숫자만큼 추출해낼 수가 있다. IE-APC 분석법은 특히 영공간(null-space) 제외한 비영공간(non-null space)에 해당하는 주성분만을 추출하는 방법으로 constraint 를 부과하여 회귀분석을 실행함으로써 식별의 문제를 해결한다. 주성분회기분석 계수는 해석이 까다롭다는 문제점이 있는데, IE-APC 는 측정된 주성분회귀계수를 역변환함으로써, 원래의 연령, 기간, 코호트를 이용해 계수를 해석할 수 있다는 장점도 있다. 따라서, IE-APC 분석법은 APC 모형이 가지고 있는 식별 문제을 해결하기 위하여, 주성분회귀분석으로 변환하여 각각의 주성분에 대한 회귀계수를 추정한 후, 다시 원래의 회귀방정식으로 역변환하는 방법이라 할 수 있다[13, 39].

IE-APC 분석법에 대한 이해를 돕기 위해 국내 갑상선암 발생률의 APC 분석 연구를 살펴보고자 한다[18]. 우리나라 갑상선암의 발생률은 1999 년 이후로 가파르게 증가하였으며[40], 전 세계에서 갑상선암의 발생률이 가장 높은 것으로 보고되고 있다[41]. 우리나라 갑상선암의 갑작스러운 발생률 증가의 원인에 대하여, 발견의 증가에 따른 과잉진단에 의한 것인지, 전리성 방사선이나 비만 등 외부 환경요인의 변화에 의한 것인지에 대해 논란이 되어 왔다. 이러한 암발생률의 변화에 대하여 직접적인 증거를 얻기 힘든 경우, APC 분석을 통해 암발생률의 증가의 원인에 대한 추론이 가능하다. 중앙암등록본부에서는 1997 년부터 2011 년까지의 총인구집단 수준(aggregated population-level)의 갑상선암 발생률을 연령, 기간, 출생코호트 각각 5 년 단위로 산출한 데이터를 이용하여 IE-APC 분석을 남녀로 나누어 시행한 결과는 <그림 1-1>, <그림 1-2>과 같다[18]. 코호트효과는 남자에서는 1932 년생부터 1977 년생까지 U-shape 의

형태를 보였으나, 기간효과와 비교하여 큰 변화가 없었으며, 여자에서는 1932 년생부터 1977 년생까지는 큰 변화 없이 상대위험도가 거의 1 에 근접하는 양상을 보였다. 반면에, 기간효과는 남녀 모두에서 1997 년부터 2011 년까지 급격하게 증가하는 양상을 보였다. 결과적으로, 우리나라 갑상선암 발생률 증가에 있어 기간효과가 큰 영향을 미친 것으로 나타났으며, 이는 갑상선암 발생률의 증가가 외부환경요인이나 비만율의 변화보다 초음파검진에 의한 발견의 증가에 주로 기인하였다는 점을 시사한다.

IE-APC 분석법에서 모수에 대한 추정치는 반드시 불편추정량(unbiased estimator)과 일치하지 않는다는 점을 명심해야 한다. 그럼에도 불구하고, IE-APC 분석법은 추정치의 분산을 최소로 만들어주기 때문에, 제약된 일반선형모델과 같은 이전의 APC 분석법에 비해 통계적효율성이 좋은 추정치를 산출한다[39]. 또한, 각 변수들의 기준점(reference)만 설정해준다면, 외부 정보나 이론에 의존하여 추가적인 제한 (constraint)을 부과하지 않고도, 강건한(robust) 추정치를 산출해낸다는 장점이 있다[42]. 실제 분석에 있어서도 5 년 단위의 나이-기간의 데이터만 있으면 분석이 가능하고, 해석하기 쉽다는 장점이 있으며, 이미 Stata 명령어[43, 44] 및 R 코드[31]가 공개되어 있어, 상대적으로 연령-기간-코호트 분석을 수행하기 쉽다.

IV. 교차분류임의효과모델(cross-classified random effects models)을 이용한 위계적(Hierarchical) APC 분석법(HAPC 분석법)

앞서 살펴본 모델은 5 년 단위의 나이-기간의 총인구집단 수준(aggregated population-level)의 데이터만으로 APC 의 각 효과를 확인할 수 있다. 그러나 기간효과와 코호트효과의 원인에 대해서는 추정만이 가능할 뿐 어떠한 기간 특성 또는 코호트 특성이 영향을 미친 것인지 실증적인 분석이 불가능하다. 연령, 기간, 코호트 외에도 공변인이나 통제 변인을 지정할 수 있다는 장점이 있어, 장기간의 건강 수준 및 건강 불평등의 원인에 대한 보다 구체적인 탐구가 가능하다[28, 35].

반복적 단면조사의 경우 국내에서는 전국단위 표본조사인 국민건강영양조사와 지역사회건강조사가 대표적인 예이다. 전국단위 표본집단을 대상으로 반복적으로 수집한 자료를 하나의 데이터셋으로 모은 통합표본자료(pooled dataset)를 분석에 사용한다. 이 경우 여러 시점에 여러 나이의 표본 응답자들로 자료가 구성되면서 이로부터 코호트를 추출해낼 수 있게 되고 APC 분석을 적용할 수 있게 된다.

<표 1>은 반복단면자료를 전형적인 구조를 보여주고 있다. IE-APC 분석에 사용된 5 년 단위 연령-기간 데이터와 세 가지 다른 점을 볼 수 있다. 우선 반복적 단면조사의 경우 기간-코호트의 셀에 표본수가 없는 경우가 있는데 이로 인해 unbalanced dataset 이라고 불린다. 이는 기존의 세대가 죽음으로 인해 조사에서 빠져나가고 새로운 세대가 점차적으로 유입되기 때문이다. 또한 반복적 단면조사자료를 이용할 경우 기간 간격(period interval)과 연령 간격(age interval)이 다를 수 있고 조정도 가능하다. 마지막으로 다수준모형의 관점에서 바라보면, 각 셀에 속한 개인이 기간과 코호트라는 상위개념에 동시에 속한 것으로 볼 수 있다. 이는 마치 다수준분석을 이용한 건강연구에서 개인이 속한 공간적인 상위 맥락인 근린특성(neighborhood characteristics)의 효과를 살펴보듯이 시간적인 측면에서 개인이 속한 상위 맥락인 기간효과의 특성 및 코호트효과의 특성의 효과를 살펴볼 수 있음을 시사한다.

Volume: 39, Article ID: e2017056
https://doi.org/10.4178/epih.e2017056

다수준 분석의 모형 중 서로 위계적 속성이 없는 두 개 이상의 상위 개념을 고려해야 할 때 사용하는 것이 교차분류임의효과모델(cross-classified random effects models, CCREMs)로서 APC 분석법에 적용하면 연령과 다른 개인 수준의 변수들의 효과를 레벨 1 의 고정효과(fixed effects)부분으로 두고 기간 효과와 코호트효과를 레벨 2 의 무작위 효과(Random effects)부분으로 모델을 설정한다[28, 35]. 따라서 HAPC 분석을 통해 개인수준에서 측정되지 않은 기간효과와 코호트효과의 무작위 변이 (random variance)를 측정할 수 있으며, 기간 특성 변수 또는 코호트 특성 변수를 모델에 추가하여 이들이 인구집단의 건강수준과 건강불평등의 추이와 변동에 미치는 영향을 설명할 수 있다[45].

이해를 돕기 위해 다음과 같은 가상의 간단한 연구의 예를 살펴보고자 한다. 연구자는 어떤 국가에서 수집한 자료를 토대로 성인 사망률 격차의 장기적인 추세를 확인하고 다음과 같은 연구 질문을 갖게 되었다. (1) 성인 사망률의 추세에 기여하는 연령효과, 기간효과, 코호트효과는 어떠한가? (2) 코호트 특성변수로서 동시에 태어난 인구수(코호트 사이즈)와 태어난 시기의 경제적 상황이 사망위험과 상관이 있는가? (3) 연령에 따른 남녀의 사망 위험의 궤적은 어떻게 다른가?

<그림 2>는 남녀간 사망률 격차에 대한 연령효과, 기간효과, 코호트효과를 사망위험의 예상확률치(predicted probabilities)로 보여준다. 세 효과 중 어느 한 효과는 다른 두 효과의 값이 평균일 때의 값이다. 즉, 맨 왼쪽의 연령효과는 모든 기간효과와 모든 코호트효과가 평균값일 때 각 연령대의 사망위험이다. 연령효과 그래프는 생애주기에 따른 사망위험을 보여주는데 연령이 늘수록 사망위험은 기하급수적으로 증가했다. 코호트는 1970 년대생들이 다른 코호트에 비해 사망위험이 가장 큰 것으로 나타났고 이후 코호트에서는 점차 격차가 줄어들고 있다. 최근으로 올수록 기간효과로 인한 사망위험은 줄어드는 것으로 나타났다.

두 번째 연구질문에 답하기 위해 위의 분석모형에 출생연도 변수와 연결된 태어난 해의 경제지표 변수와 코호트 사이즈 변수를 넣고 재분석하였다. <표 2>의 결과를 살펴보면, 연구질문 (1)을 답하기 위해 앞서 설정한 모델에서 임의효과 부분의 분산은 0.345 이다. 이에 두 변수를 모델에 포함시킨 결과 모두 사망위험의 증가와 유의한 연관이 있는 것으로 나타났다. 즉, 태어난 시기에 경제상황이 좋을수록 이들의 향후 사망위험 감소와 연관이 있었다. 그러나 코호트의 크기가 클수록, 즉 같은 시기에 태어난 인구수가 클수록 향후 높은 사망위험과 연관이 있었다. 태어날 때의 경제상황은 코호트 분산의 60.3%[((0.345−0.137)/0.345)*100]를, 코호트 사이즈는 35.7%[((0.345−0.220)/0.345)*100]를 설명하고 있다.

세 번째 연구질문에 대답하기 위해 원래 분석모형에 연령과 성별의 교호작용 변수를 넣고 분석한 결과를 바탕으로 <그림 3>과 같은 그래프를 그렸다. 연령에 따른 사망위험의 궤적이 성별에 따라 다름을 보여주고 있으며 남자가 모든 연령에서 여성보다 사망위험이 높으나 그 차이는 연령이 높아질수록 감소하는 것으로 나타났다.

따라서 HAPC 모델을 사용한 연구는 건강수준 및 건강불평등의 장기간에 걸친 인구집단의 구성 변화와 사회맥락의 변화를 동시에 고려하여 측정할 수 있을 뿐 아니라 건강수준 및 건강불평등을 결정하는 해당 구성효과와 맥락효과의 요인이 무엇인지 밝히는데 도움이 되는 방법이다.

V. 가속종단패널데이터를 이용한 위계적 APC-성장곡선모델(Hierarchical APC-Growth Curve model)

이전의 두 모델이 각각 데이터 수집 시점에서 여러 연령의 구성원을 포함하는 데이터구조를 활용하여 진짜 코호트를 대리하도록 코호트를 합성하는 식이었다. 따라서 인과관계 또한 합성된 코호트들의 시간에 따른 변화가 진짜 코호트들의 연령에 따른 변화처럼 보여질 수 있을 때 성립된다. 즉, 코호트의 구성이 이민 등의 영향이 배제되어서 시간에 따라 크게 변하지 않고 샘플의 크기가 커야 한다는 조건이 있다[13]. 그러나 최근 들어 여러 실제 코호트들을 여러 시점에서 동시에 추적하여 자료를 축적하는 가속종단패널데이터(accelerated longitudinal panel data)를 이용 가능하게 되면서 진짜 코호트로부터 정보를 얻는 것이 가능해졌다. 패널자료 사용을 위해 역시 다수준 분석법을 적용하여 코호트를 레벨 2 에, 시간에 따라 반복되는 개인의 측정값을 레벨 1 에 설정하고 분석한다.

성장곡선모델(growth curve model)을 사용하면 코호트 내의 연령에 따른 변화(intra-cohort variations)와 코호트간의 차이(inter-cohort variations)를 밝혀냄으로써 인구집단간의 생애주기에 걸친 불평등의 메커니즘을 밝혀내는데 기여할 수 있다. 다시 말하면, 이 모델을 통해 독립적인 연령효과와 코호트효과에 대해 다음과 같은 연구 질문에 대답할 수 있다. 첫째, 고령화 과정에서 코호트간 변이(inter-cohort variations)가 존재하는가? 즉 나이에 따른 변화가 코호트에 따라 다른가? 만일 이 질문에 그렇다고 대답할 수 있으면 코호트효과를 고려하지 않은 기존의 고령연구들의 나이에 따른 변화는 연령효과와 코호트효과가 교란된 결과일 수 있다. 둘째, 고령화 과정에서 코호트 내 변이(intra-cohort variations)가 존재하는가? 즉, 각 코호트 내에 사회경제적 지위와 같은 특성에 따라 나이에 따른 노화궤적이 다르게 나타나는지를 묻는다. 첫 번째 질문은 코호트를 동질한 성격의 그룹으로 간주하는 반면 두 번째 질문에서는 더 나아가 코호트 내의 이질성(within-cohort heterogeneity)을 확인하는 것이다. 세 번째, 코호트 내 고령화로 인한 변화에 있어 코호트간 변이(inter-cohort variations)가 존재하는가? 이 질문은 코호트 내 및 코호트간의 이질성을 확인하는 것으로 개인의 고령화가 사회적, 역사적, 역학적 맥락에 따라 어떻게 변화되어 왔는지를 알려준다.

이해를 돕기 위해 분석결과의 예를 들어보고자 한다. 이 모델을 사용한 분석한 결과가 <그림 4-1>과 같이 나왔다고 가정해보자. 연구 결과는 우선 최근 코호트로 올수록 주관적 건강수준의 평균값이 높은 것으로, 코호트간 변화가설을 뒷받침하고 있다. 두 번째 연구 질문에 대한 답으로 코호트 내 불평등 가설을 지지하는 결과로, 코호트 내의 남녀 간의 주관적 건강수준에 있어서 불평등이 지속되고 있음을 보여주고 있다.

<그림 4-2>는 세 번째 연구 질문에 대한 답의 예시를 보여준다. 수입에 따른 코호트 내의 주관적 건강의 차이가 코호트 간에도 존재하는가를 알아 보기 위해 연령, 코호트, 수입 간의 3 원 교호효과(3-way interaction) 분석을 실시했다. <그림 4-2>은 주관적 건강수준에 있어서 수입 수준에 따른 코호트간 차이의 변화가 나이에 따라 변화하고 있음을 보여주고 있는 것으로 코호트에 따라서 수입에 따른 주관적 건강의 성장률(growth rates)의 차이가 존재하고 그 차이가 증가하고 있다고 말할 수 있다.

여러 코호트를 여러 시점에 측정한 데이터를 사용하여 성장곡선모델을 설정하고자 할 때는 일반적인 종단면 연구와는 다르게 고려해야 하는 사항들이 있다. 우선, 연령에 따른 궤적은 각기

다른 코호트마다 시작과 끝이 다르기 때문에 코호트 간 비교를 하려면 코호트 간에 겹쳐지는 일부분을 살펴봐야 하는데 이는 연령효과와 코호트효과를 교란하는 셈이 된다. 이를 해결하기 위해 코호트별로 연령을 센터링하여 절편의 해석을 돕는 한편, 추정값을 안정시키고, 코호트에 걸친 연령 평균의 조직적인 변이로 인한 편향을 방지할 수 있게 된다[13]. 두 번째로 이 모델은 인접 코호트 간에 겹치는 생애주기의 부분에 대한 유의성 테스트를 할 수 있다. 조사 시점이 많아질수록 인접 코호트 간에 겹치는 부분이 증가하게 되고 이는 통계적 파워가 증가하게 된다. 이럴 경우 연령과 코호트 간의 교란은 점차 줄어들고 고령 궤적의 코호트별 차이가 비교 가능하게 된다[13].

이 모델의 한계는 기간효과를 고려하지 않는다는 것이다. 그 첫 번째 이유는 상대적으로 긴 기간 축적된 합성 코호트 자료와는 달리 종단면 연구는 조사 기간이 길지 않기 때문이다. 따라서 기간효과는 특히 고령연구의 경우 무시할 만하거나 생략된 것으로 간주하고 분석한다. 두 번째 이유는 가속종단패널데이터에서 기간효과를 분리해서 추정하는 것이 불가능하다는데 있다 성장곡선모델에서는 레벨 1 분석은 연구 질문에 따라 연령 또는 기간을 포함하는 것은 가능하지만, 한 개인의 관점에서 보면 연령과 기간이 동일하기 때문에 두 측면을 동시에 고려할 수 없다.

소결하면, APC 분석법은 사회적 변화, 질병과 노쇠 및 인구집단의 불평등의 변화에 대한 지속되어 온 연구질문들에 답할 수 있는 방법이다. 본 논문에는 지면의 한계상 싣지 않았으나 코호트효과를 무시한 연령과 기간에만 의존한 기존의 장래 추세 예측보다 나은, 연령효과, 기간효과, 코호트효과의 각 변화의 추세에 기반한 향후 추세 예측 연구도 가능하다[13, 46].

APC 분석법은 질 높은 데이터의 축적과 분석 모델의 향상으로 식별 문제를 창의적으로 해결하는 방향으로 계속적으로 발전해왔고 지금도 발전되고 있다. 이 과정에서 식별 문제에 대해 연구자마다 다른 해답을 제시하면서 같은 일관되지 않은 연구결과가 발표되면서 식별 문제를 해결할 수 있는지, 더 나은 방법은 없는지에 논쟁이 벌어지기도 한다. 따라서 상기에서 소개한 방법론이 최후의 APC 분석법이라고 할 수 없다. 조지 박스(George E. P. Box) 교수가 언급한 유명한 말처럼 여느 통계학적 모델이 그렇듯이 현상을 모두 그려낼 수 없다는 점에서 "틀렸다"고 할 수 있으나 어떤 모델들은 더 나은 통계적 특성으로 인해 현상의 "이해를 돕고 유용"하다고 할 수 있다[47]. 현재도 APC 분석법이 가지고 있는 식별문제를 해결하기 위한 여러 방법들이 개발되고 있다(예를 들어, [48]). 하지만 식별문제를 손쉽게 해결하는 마법의 해결책은 없기 때문에 본 연구에서 제시된 방법들은 자료와 분석모델에 대한 충분한 이해를 바탕으로 주의깊게 사용될 필요가 있다.

VI. 결론: 국내에서의 APC 연구의 미래

칼 만하임은 급격한 변동을 겪는 사회일수록 코호트간의 역사적 ·문화적 경험의 차이가 크기 때문에 세대격차도 심화될 것이라고 주장했다[9]. 이를 분석적 측면에서 보면 그런 사회일수록 코호트효과의 편차도 상당히 클 것이라고 예상할 수 있다. 우리나라는 해방 이후 단기간의 사회경제적 변동을 겪은바, 이러한 APC 연구를 통해 한국인들의 고령화의 궤적이 어떻게 다른지, 기간효과와 코호트효과는 어떻게 변해왔으며, 한국인의 생애에 걸친 건강 및 건강불평등에 기여하는 생물학적, 역사적, 사회구조적 요인은 무엇인지 이해할 수 있을 것이다.

국내의 현황을 살펴볼 때, 데이터의 측면에서는 위에서 언급한 세 가지 방법론에 요구되는 형태의 보건의료 데이터들이 여럿 존재한다. IE 를 이용한 사망률 또는 암을 포함한 만성질환 연구는 주로

Volume: 39, Article ID: e2017056
https://doi.org/10.4178/epih.e2017056

통계포탈(https://kosis.kr) 등에서 구득이 가능하고, HAPC 분석을 위한 반복적 횡단자료로는 대표적으로 국민건강영양조사(1998 년부터 조사 시작)와 지역사회건강조사(2008 년부터 조사 시작)가 있고, HAPC-Growth Curve Analysis 를 위해서는 한국복지패널(2006 년부터 조사 시작), 한국의료패널(2008 년부터 조사 시작), 고령화패널(2006 년부터 조사 시작) 또는 그밖에 공단 또는 건강보험심사평가원에서 제공하는 대규모 데이터베이스 등도 사용 가능하다. 따라서 연구자들은 APC 분석법을 적용하여 건강수준 및 건강행태, 건강불평등의 추세 분석 및 고령화, 만성질환, 생애주기 연구 등에 있어서 크게 기여할 여지가 있다.

나아가 APC 연구 결과를 통해서 향후 보건의료 정책에 있어서도 기간 또는 연령에 따른 정책뿐 아니라 코호트에 특정화된 정책들도 고려되어야 할 필요가 있다. 예를 들어, 태어난 코호트의 크기에 따라 교육, 보건의료, 복지 영역의 필요 자원 및 서비스 수요를 예측하고 정책적으로 대비한다든지, 경제위기 시기에 태어나는 코호트에 추가적인 생애 초기 출산 및 양육서비스를 제공하는 등의 정책적 관점이 요구될 것이다.

Reference

1. Kwon JW, Song Y-m, soon Park H, Sung J, Kim H, Cho S-i. Effects of age, time period, and birth cohort on the prevalence of diabetes and obesity in Korean men. Diabetes care. 2008;31(2):255-60.
2. Jhun HJ, Kim H, Cho S-I. Time trend and age-period-cohort effects on acute myocardial infarction mortality in Korean adults from 1988 to 2007. Journal of Korean Medical Science. 2011;26(5):637-41.
3. Choi Y, Kim Y, Park SK, Shin H-R, Yoo K-Y. Age-period-cohort analysis of female breast cancer mortality in Korea. Breast Cancer. 2006;13(3):266-71.
4. Lee JY, Kim SH. Suicide in South Korea-age-period-cohort analysis, 1983-2003. Korean J Sociol 2010;44: 63-94 (Korean).
5. Lee HA, Park H. Trends in ischemic heart disease mortality in Korea, 1985-2009: an age-period-cohort analysis. Journal of Preventive Medicine and Public Health. 2012;45(5):323.
6. Park C, Jee YH, Jung KJ. Age–period–cohort analysis of the suicide rate in Korea. Journal of affective disorders. 2016;194:16-20.
7. Seo JY, Choi S, Choi B, Ki M. Age-period-cohort analysis of hepatitis A incidence rates in Korea from 2002 to 2012. Epidemiology and health. 2016;38.
8. Jee YH, Cho S-i. Age-period-cohort analysis of smoking prevalence among young adults in Korea. Epidemiology and health. 2016;38.
9. Mannheim K. The Problem of Generation. P. Kecskemeti. Essays on the Sociology of Knowledge. 1952.
10. Ryder NB. The cohort as a concept in the study of social change. American Sociological Review. 1965:843–61.
11. Weekly Dong-A. Disable students of admission year 1994. Weekly Dong-A; 2000 Dec 14. Seoul: Weekly Dong-A; 2000, p. 48-49 (Korean).
12. Glenn ND. Cohort analysis: Sage; 2005.
13. Yang Y, Land KC. Age-period-cohort analysis. Chapman & Hall/CRC Interdisciplinary Statistics Series doi. 2013;10(1201):b13902.
14. Yang Y. Is old age depressing? Growth trajectories and cohort variations in late-life depression. Journal of health and social behavior. 2007;48(1):16-32.
15. Suzuki E. Time changes, so do people. Social Science & Medicine. 2012;75(3):452-6.
16. Williams DR. The health of US racial and ethnic populations. The Journals of Gerontology Series B: Psychological Sciences and Social Sciences. 2005;60(Special Issue 2):S53-S62.
17. Sung H, Rosenberg PS, Chen W-Q, Hartman M, Lim W-y, Chia KS, et al. Female breast cancer incidence among Asian and Western populations: more similar than expected. JNCI: Journal of the National Cancer Institute. 2015;107(7).
18. Oh CM, Jung KW, Won YJ, Shin A, Kong HJ, Lee JS. Age-period-cohort analysis of thyroid cancer incidence in Korea. Cancer research and treatment: official journal of Korean Cancer Association. 2015;47(3):362.
19. Bray F, Richiardi L, Ekbom A, Pukkala E, Cuninkova M, Møller H. Trends in testicular cancer incidence and mortality in 22 European countries: continuing increases in incidence and declines in mortality. International Journal of Cancer. 2006;118(12):3099-111.
20. Hobcraft J, Menken J, Preston S. Age, period, and cohort effects in demography: a review. Cohort Analysis in Social Research: Springer; 1985. p. 89-135.
21. Barker DJ. In utero programming of chronic disease. Clinical science (London, England: 1979). 1998;95(2):115-28.

https://doi.org/10.4178/epih.e2017056

22. Fogel RW, Costa DL. A theory of technophysio evolution, with some implications for forecasting population, health care costs, and pension costs. Demography. 1997;34(1):49-66.
23. Crimmins EM, Reynolds SL, Saito Y. Trends in Health and Ability to Work Among the Older Working-Age Population. The Journals of Gerontology: Series B. 1999;54B(1):S31-S40. doi: 10.1093/geronb/54B.1.S31.
24. Freedman VA, Martin LG. Understanding trends in functional limitations among older Americans. American journal of public health. 1998;88(10):1457-62.
25. Mason KO, Mason WM, Winsborough HH, Poole WK. Some methodological issues in cohort analysis of archival data. American sociological review. 1973:242-58.
26. Kupper LL, Janis JM, Karmous A, Greenberg BG. Statistical age-period-cohort analysis: a review and critique. Journal of chronic diseases. 1985;38(10):811-30.
27. Mason WM, Fienberg SE. Introduction: Beyond the identification problem. Cohort analysis in social research: Springer; 1985. p. 1-8.
28. Yang Y, Land KC. A mixed models approach to the age-period-cohort analysis of repeated cross-section surveys, with an application to data on trends in verbal test scores. Sociological Methodology. 2006;36(1):75–97.
29. Reither EN, Masters RK, Yang YC, Powers DA, Zheng H, Land KC. Should age-period-cohort studies return to the methodologies of the 1970s? Social Science & Medicine. 2015;128:356-65.
30. Yang Y, Fu WJ, Land KC. 2. A Methodological Comparison of Age-Period-Cohort Models: The Intrinsic Estimator and Conventional Generalized Linear Models. Sociological methodology. 2004;34(1):75-110.
31. Tu Y-K, Krämer N, Lee W-C. Addressing the identification problem in age-period-cohort analysis: a tutorial on the use of partial least squares and principal components analysis. Epidemiology. 2012;23(4):583-93.
32. Holford TR. The estimation of age, period and cohort effects for vital rates. Biometrics. 1983:311-24.
33. Heuer C. Modeling of time trends and interactions in vital rates using restricted regression splines. Biometrics. 1997:161-77.
34. Yang Y, Schulhofer-Wohl S, Fu WJ, Land KC. The Intrinsic Estimator for Age-Period-Cohort Analysis: What It Is and How to Use It 1. American Journal of Sociology. 2008;113(6):1697-736.
35. Yang Y, Land KC. Age–period–cohort analysis of repeated cross-section surveys: fixed or random effects? Sociological methods & research. 2008;36(3):297-326.
36. Dassonneville R. Questioning generational replacement. An age, period and cohort analysis of electoral volatility in the Netherlands, 1971–2010. Electoral Studies. 2013;32(1):37-47.
37. Jeon SY, Reither EN, Masters RK. A population-based analysis of increasing rates of suicide mortality in Japan and South Korea, 1985–2010. BMC public health. 2016;16(1):356.
38. Reither EN, Hauser RM, Yang Y. Do birth cohorts matter? Age-period-cohort analyses of the obesity epidemic in the United States. Social science & medicine. 2009;69(10):1439-48.
39. Yang Y, Schulhofer-Wohl S, Fu WJ, Land KC. The intrinsic estimator for age-period-cohort analysis: What it is and how to use it. American Journal of Sociology. 2008;113(6):1697-736.
40. Oh C-M, Won Y-J, Jung K-W, Kong H-J, Cho H, Lee J-K, et al. Cancer statistics in Korea: incidence, mortality, survival, and prevalence in 2013. Cancer research and treatment: official journal of Korean Cancer Association. 2016;48(2):436.
41. Ferlay J, Soerjomataram I, Dikshit R, Eser S, Mathers C, Rebelo M, et al. Cancer incidence and mortality worldwide: sources, methods and major patterns in GLOBOCAN 2012. International journal of cancer. 2015;136(5).

Volume: 39, Article ID: e2017056
https://doi.org/10.4178/epih.e2017056

42. Land KC, Zang E, Fu Q, Guo X, Jeon SY, Reither EN. Playing with the rules and making misleading statements: a response to luo, hodges, winship, and powers. American journal of sociology. 2016;122(3):962-73.
43. Rutherford MJ, Lambert PC, Thompson JR. Age-period-cohort modeling. Stata Journal. 2010;10(4):606.
44. Schulhofer-Wohl S, Yang Y. APC: Stata module for estimating age-period-cohort effects. Statistical Software Components. 2006.
45. Yang Y. Aging, Cohorts, and Methods. In: George RHBLK, editor. Aging and the Social Sciences: Elsevier Inc.; 2011. p. 17-30.
46. Reither EN, Olshansky SJ, Yang Y. New forecasting methodology indicates more disease and earlier mortality ahead for today's younger Americans. Health Affairs. 2011:10.1377/hlthaff. 2011.0092.
47. Box GE. Science and statistics. Journal of the American Statistical Association. 1976;71(356):791-9.
48. Fosse E, Winship C. Nonparametric Bounds of Age-Period-Cohort Effects. 2016.

Volume: 39, Article ID: e2017056
https://doi.org/10.4178/epih.e2017056

<표 1> 반복단면자료를 전형적인 구조: 연령−코호트에 따른 응답자 수(가상의 데이터)

	Year								
Cohort	2008	2009	2010	2011	2012	2013	2014	2015	Total
1930	12	5	1	0	0	0	0	0	18
1935	31	26	28	12	0	0	0	0	97
1940	51	37	37	26	11	5	0	0	167
1945	107	69	59	49	38	26	10	0	358
1950	128	106	91	82	52	37	19	5	520
1955	150	116	103	99	82	73	34	21	678
1960	179	128	134	123	115	93	59	38	869
1965	153	160	184	178	129	109	94	76	1083
1970	110	130	154	164	158	154	152	100	1122
1975	93	84	111	116	123	120	125	105	877
1980	42	42	83	65	107	96	110	143	688
1985	15	16	31	54	61	87	108	171	543
1990	0	0	0	35	52	77	90	101	355
1995	0	0	0	0	0	0	0	18	18

Volume: 39, Article ID: e2017056
https://doi.org/10.4178/epih.e2017056

<표 2> 코호트 특성 변수의 추정치(estimates)와 분산(variance)

	β	Var
Covariate adjusted	-	0.345
GDP at birth	-0.458***	0.137
Cohort size	0.598***	0.220

***p<0.001

Volume: 39, Article ID: e2017056
https://doi.org/10.4178/epih.e2017056

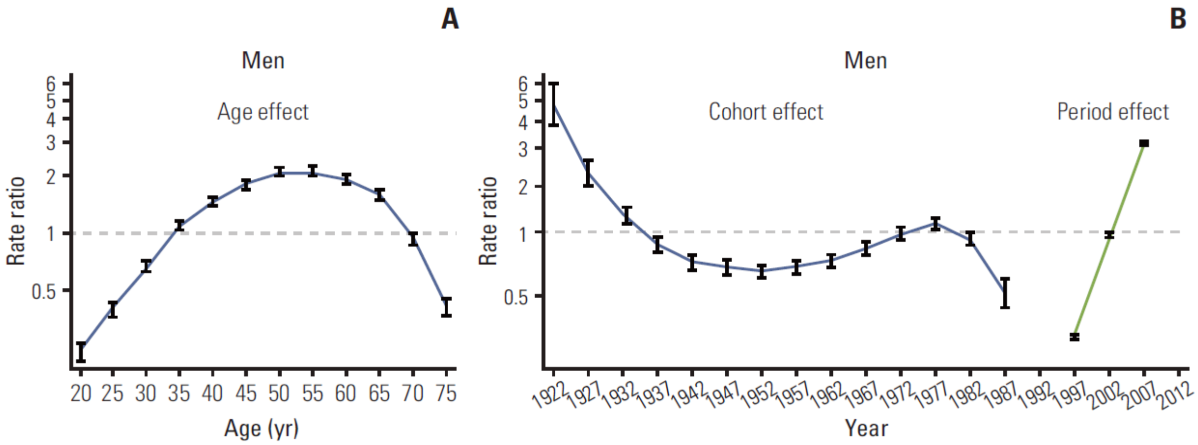

<그림 1-1> 한국 성인남성의 갑상선 암 IE-APC 분석 (출처: Oh, Jung (18)), 재인용)

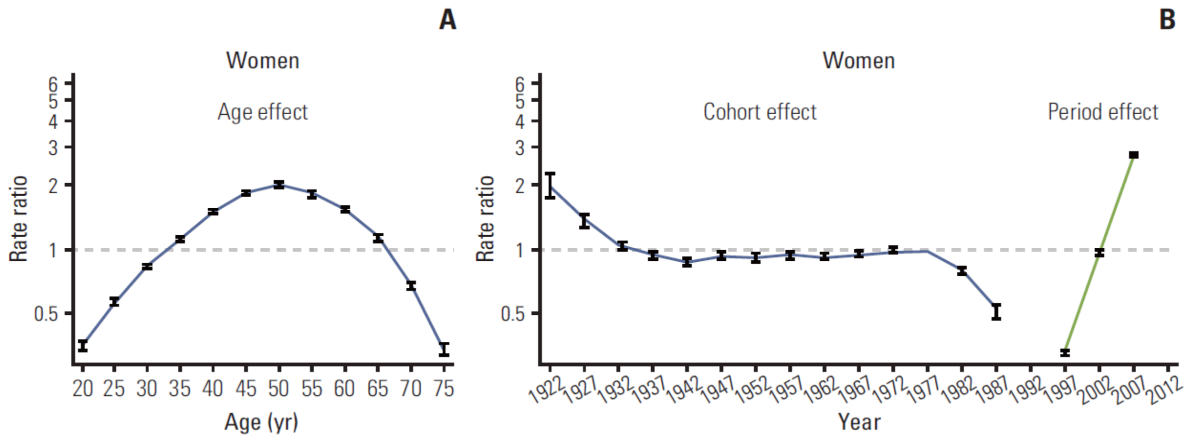

<그림 1-2> 한국 성인여성의 갑상선 암 IE-APC 분석 (출처: Oh, Jung (18)), 재인용)

Volume: 39, Article ID: e2017056
https://doi.org/10.4178/epih.e2017056

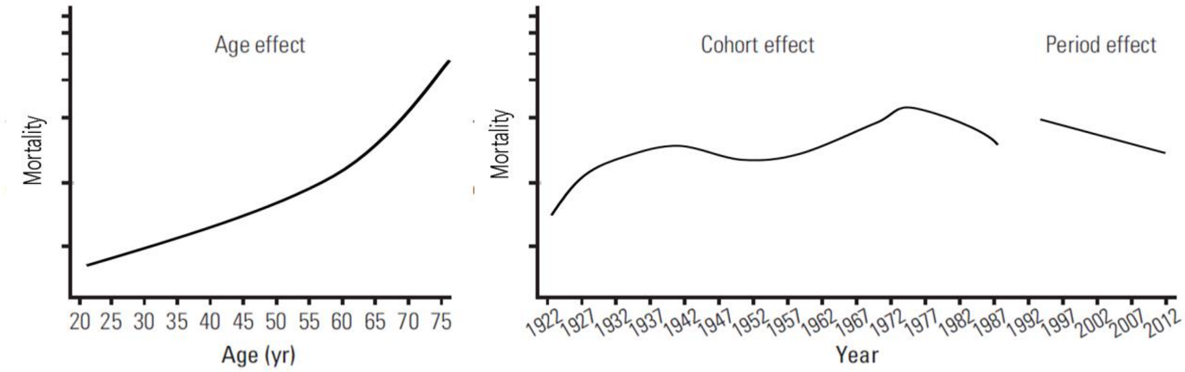

<그림 2> 성인 사망률 추세의 HAPC 분석결과 예시

Volume: 39, Article ID: e2017056
https://doi.org/10.4178/epih.e2017056

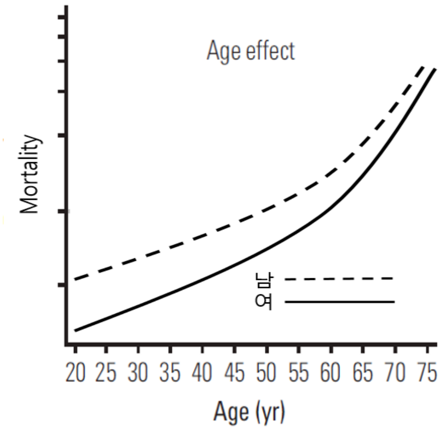

<그림 3> 연령에 따른 남녀의 사망 위험의 궤적

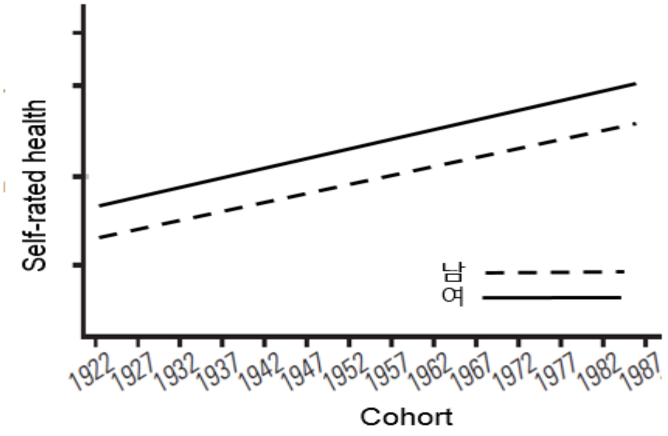

<그림 4-1> 코호트에 따른 자가보고건강의 남녀간 격차

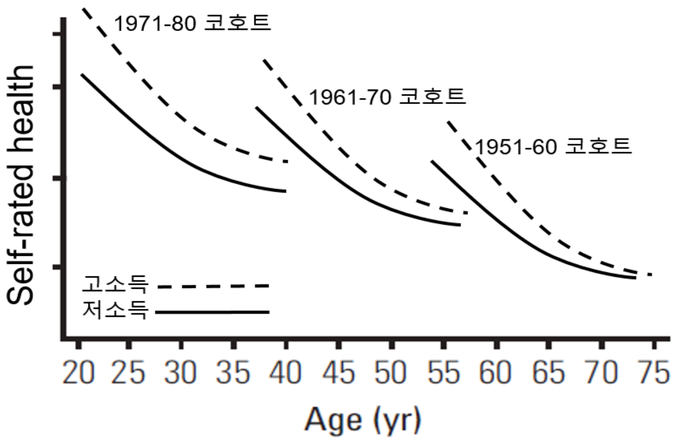

<그림 4-2> 코호트별 자가보고건강의 연령성장궤적에서의 수입그룹간 격차
